# Automatic detection of exonic splicing enhancers (ESEs) using SVMs

**DOI:** 10.1186/1471-2105-9-369

**Published:** 2008-09-10

**Authors:** Britta Mersch, Alexander Gepperth, Sándor Suhai, Agnes Hotz-Wagenblatt

**Affiliations:** 1 Department of Molecular Biophysics, German Cancer Research Center (DKFZ), Im Neuenheimer Feld 580, 69120, Heidelberg, Germany; 2 Honda Research Institute Europe GmbH, Carl-Legien-Straße 30, 63073, Offenbach/Main, Germany

## Abstract

**Background:**

Exonic splicing enhancers (ESEs) activate nearby splice sites and promote the inclusion (vs. exclusion) of exons in which they reside, while being a binding site for SR proteins. To study the impact of ESEs on alternative splicing it would be useful to have a possibility to detect them in exons. Identifying SR protein-binding sites in human DNA sequences by machine learning techniques is a formidable task, since the exon sequences are also constrained by their functional role in coding for proteins.

**Results:**

The choice of training examples needed for machine learning approaches is difficult since there are only few exact locations of human ESEs described in the literature which could be considered as positive examples. Additionally, it is unclear which sequences are suitable as negative examples. Therefore, we developed a motif-oriented data-extraction method that extracts exon sequences around experimentally or theoretically determined ESE patterns. Positive examples are restricted by heuristics based on known properties of ESEs, e.g. location in the vicinity of a splice site, whereas negative examples are taken in the same way from the middle of long exons. We show that a suitably chosen SVM using optimized sequence kernels (e.g., combined oligo kernel) can extract meaningful properties from these training examples. Once the classifier is trained, every potential ESE sequence can be passed to the SVM for verification. Using SVMs with the combined oligo kernel yields a high accuracy of about 90 percent and well interpretable parameters.

**Conclusion:**

The motif-oriented data-extraction method seems to produce consistent training and test data leading to good classification rates and thus allows verification of potential ESE motifs. The best results were obtained using an SVM with the combined oligo kernel, while oligo kernels with oligomers of a certain length could be used to extract relevant features.

## Background

**Figure 1:**
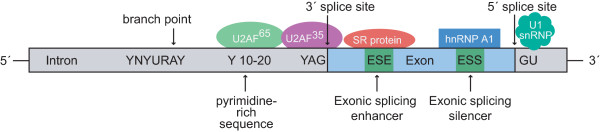
**Recognition signals and proteins for splicing**. The consensus sequences which occur in most of the eukaryotes are shown. Y = pyrimidine, R = purine, N = any nucleotide.

In eukaryotes, after transcription from DNA to messenger RNA (mRNA), the mRNA is initially present as a precursor messenger RNA (pre-mRNA). This pre-mRNA still comprises the exons and introns of the gene. At this stage it is not known which exons will eventually be included into the mature mRNA. This decision is taken during a process called splicing. Then, the introns are cut out and the exons are reconnected in different ways to yield various mRNAs. The signals which are necessary to define the splice sites are located at the exon/intron boundaries and in the introns, see Figure 1. Especially the AG- and GU-dinucleotides at the ends of introns are highly conserved. In some proteins, the exons are joined in the order in which they appear on the pre-mRNA. However, mostly this is not the case. Different variants of a protein can occur when single exons are skipped or if two splice sites are present in an exon from which only one is used. This is called alternative splicing. Some of the exons are present in every mRNA (constitutive exons), others are alternatively spliced, and thus only present in some of the transcripts. The resulting mRNAs are then translated into proteins which can differ extremely in their function.

The splicing process involves a series of biochemical reactions which are catalyzed by the spliceosome, a complex of small nuclear ribonucleo-proteins (snRNPs). Additionally, there are both cis-acting sequence elements as well as trans-acting elements involved. It is known that splicing is partly promoted by additional sequences which are embedded in exons. Depending on whether the sequence elements assist or inhibit the splicing process, they are called exonic splicing enhancers or exonic splicing silencers. Exonic splicing enhancers (ESEs) are usually purine-rich sequences that bind members of the SR protein family (see Figure 1). SR proteins are Serine/Arginine-rich proteins which can bind to the RNA and are required during alternative splicing to select which splice site should be used [1-3]. Their binding to these elements enhances or prevents the steric association of the 5'-and 3'-splice sites and consequently has an essential function in exon definition.

Exonic splicing enhancers are not easy to identify because known ESEs consist of quite common motifs of only 6–8 bases which occur at many positions in the genome. Therefore, not every such sequence indicates a binding site. A well-known group of ESEs are the purine-rich enhancers containing repeated GAR (GAG and GAA) motifs. Several studies have shown that many sequences can function as ESEs. The employment of functional systematic evolution of ligands by exponential enrichment (SELEX) is an important tool for ESE identification [4-9]. The identified motifs are short (6–8 nt) and degenerate.

In [10], ab initio computational approaches for the identification of ESE motifs were employed. RESCUE-ESE identified motifs of length six which are over-represented in exons with non-consensus splice sites ("weak exons") as well as in exons versus introns. In [11], a similar approach to identify octamers over-represented in internal non-coding exons versus unspliced pseudo-exons and the 5' untranslated regions of intronless genes was utilized. Both methodologies resulted in motifs that function as ESEs, as the authors showed experimentally in several cases. Until now, the bioinformatics tools for detecting exonic splicing enhancers are often based on position-weight matrices (PWMs) which were constructed from experimentally verified motifs of ESE sequences. Because of the shortness of ESEs, the use of PWMs leads to a high amount of false positive predictions. Of course, a high amount of false positive predictions is not desirable as it makes the classification less useful for biologists. Every potential positive prediction needs experimental verification. However, a high number of experiments examining all predictions will waste time, money and material. In order to use the predictions to guide experiments, the false positive prediction rate has to be reduced. Examples for pattern matching based programs are ESEfinder [12] and SEE ESE [13].

In order to analyze alternative splicing it is necessary to have an accurate detection method for ESEs. Loss or disruption of ESEs can result in a changed transcript, potentially even be responsible for diseases [2].

Here, we report on a new approach using support vector machines (SVMs, [14-16]) with special sequence based kernels to perform the classification of exonic splicing enhancer sequences and our own strategy for the generation of the data sets for training and testing the classifiers.

## Results and discussion

### Design of positive and negative data sets

A problem for detecting ESEs is the selection of suitable sequences for training and testing the SVMs. There are only few exact locations and motifs of ESEs described in the literature. Therefore, we tested two different data generation methods that are presented below.

#### Neutralized data

The idea behind the data set was obtained from [17]. The data set contained 1000 randomly chosen protein-coding exons with length ranging from 100 to 300 bases from the Vega database [18], which we assume to contain exonic splicing enhancers. From these exons, a set of 1000 negative sequences was created using a mechanism called neutralization [17]. The negative training sequences were generated randomly, but still coded for the same amino acid sequence and maintained the overall composition of the original exons. That is, the codon usage should be preserved as well as the frequencies of dinucleotide occurrences. According to the authors, using this training data resulted in features which performed some function independent of the protein-coding function of exons and can thus be used to discriminate between the original and the neutralized data set. 200 cycles of neutralization were used leading to a mean difference of 73% between the exons and the neutralized counterparts. As described in [17], we examined the dinucleotide composition before and after neutralization and found the frequencies changed only minimally. For a more detailed description of the neutralization procedure see the Methods section or the original literature [17].

#### Motif-oriented data

For generating a second data set, we developed a motif-oriented data-extraction method. Sequences were extracted locally around experimentally or theoretically determined ESE patterns, where we used the 238 hexamers identified by RESCUE-ESE [10]. We assumed that the sequence surrounding an ESE pattern can help to detect them in exons. This is reasonable because of the fact that ESEs are located in the vicinity of other binding sites for splicing proteins or ESEs itself as well as in the vicinity of splice sites [19,20]. As previously mentioned, the ESE patterns are quite short and not every such sequence indicates a binding site. Therefore, positive examples were restricted by heuristics based on known properties of ESEs:

• Location in the vicinity of splice sites [19,20]

• Presence in an exon with a non-consensus splice site (''weak exon'') [3,10]

• Location in a single-stranded region [21]

In summary, these criteria led to consistent positive examples, from which local features could be extracted. We obtained 1835 sequences which met the above-mentioned criteria and could thus be used as positive training examples. An advantage of this method is that the classification problem is simplified by the introduction of biological a priori knowledge. A disadvantage is that the new method can only be used for ESEs with known consensus sequences.

The negative examples were extracted from longer exons using the same extraction method as used for positive examples, positioning the "ESE motif" in the center and extracting the surrounding sequence. An advantage is that these sequences have the same background distribution of the four bases as in the positive examples. Due to the fact that ESEs are only active in the vicinity of the splice sites, ESE motifs in the middle of long exons should not have any ESE-activity and can be used as negative examples. Additionally, we used only ESE motifs which are located in double-stranded regions. These were identifiable based on the fact that small energy values [see Methods] label a substring as double stranded (*EF*_*a,b *_< 0.3). This increased the possibility of reliable negative examples. A large set of sequences met these criteria and as such we undersampled the negative class by randomly selecting 3000 sequences.

### SVM scenario

An *L*_1_-norm *soft margin support vector machine *(SVM) was applied using special sequence based kernels, the combined oligo kernel [22] and the locality improved kernel [15,23]. The *combined oligo kernel *counts matching oligomers up to a certain length with an adjustable degree of positional uncertainty. This uncertainty is realized using the smoothing parameters *σ*_1_,...,*σ*_*κ *_of the Gaussian in the combined oligo kernel function [see Methods]. The *locality improved kernel *counts matching nucleotides and considers local correlations within local windows of length 2*l *+ 1 [see Methods]. For comparison, a Markov chain model was implemented.

### Results for neutralized data

The data for training and testing the SVM classifier consisted of 1000 positive examples, the exons, and 1000 negative examples, their neutralized counterparts. We performed 50 trials with different random partitions of the data into training and test sets.

#### Adaptation of the parameters of the SVM kernel

In the training phase the parameters of the used kernel had to be adapted. In this case, the *oligo kernel *[see Methods] was employed. A grid search was used in a 5-fold cross-validation scenario for determining the optimal values of the smoothing parameter *σ *∈ {*i *| 1 ≤ *i *≤ 10} of the oligo kernel as well as the regularization parameter *C *∈ {0.002·*i *| 1 ≤ *i *≤ 10} of the SVM.

#### Classification performance

Training an SVM classifier using the oligo kernel resulted in an accuracy of about 95%. This was quite high and unexpected. To analyze the classification performance further, a number of different data sets were used for testing. These consisted of coding exons which were not in the training set, non-coding exons, introns and intergenic regions. From each of these sets, the negative examples were either generated using the neutralization procedure (even if the sequences were not protein-coding) or a randomization process. Randomization generates a random counterpart of the original sequence while maintaining mononucleotide and dinucleotide composition [17]. We expected that for the coding exons as well as for the non-coding exons the classification rates would be good for both types of negative examples. This would have shown that the classifier had extracted exon-specific signals from the original training data from which some were general to both coding and non-coding exons. In contrast, for introns and intergenic regions, we expected that the accuracy would be poor due to the fact that these sequences do not contain exon-specific signals. Using the new data sets as test data for the trained classifier, we obtained accuracies as shown in Table 1. The classification performance for the sets with randomized negative examples were poor, approximately at chance level. In contrast, the performance for the neutralized negative examples was good for all additional data sets mentioned above. This suggested that the classifier learns neutralization-specific features from the data, but not exon-specific features. Thus, it seemed as if the neutralization procedure produced artifacts which could be exploited by the classifier. We can conclude this because the classification performance for introns and intergenic regions should have been poor as well since none of the underlying features contained in exons occur in these sequences. Therefore, at least for exons which were not in the original data set the classifier should have been able to distinguish between them and the randomized counterparts.

**Table 1:** Results of tests for neutralization procedure

	randomized negatives	neutralized negatives
protein coding exons not in training set	53.3%	95.8%
non-coding exons	51.11%	89.58%
introns	53.72%	83.96%
intergenic regions	52.06%	87.27%

This table gives the accuracies for the classifications with the external test sets. As "positive" examples, protein coding exons not in the training set, non-coding exons, introns and intergenic regions are used. As negative examples, both randomized counterparts and neutralized counterparts to each of the positive sets are used.

### Results for motif-oriented data

Since the results with the neutralized negative examples were not very promising, we developed the motif-oriented data-extraction scheme as described before. For the exact mechanism of generating the data sets, please refer to the Methods section. The data for training and testing the SVM classifier consisted of 1835 positive examples and 3000 negative examples. We performed 50 trials with different random partitions of the data into training and test sets.

#### Adaptation of the parameters of the SVM kernels

In the training phase the parameters of the kernels had to be adapted to the given task of classifying ESEs. For the adaptation of the combined oligo kernel, we used the recently proposed gradient-based optimization of the kernel-target alignment [24] in a 5-fold cross-validation scenario for the parameters *σ*_1_,..., *σ*_*κ*_. In our experiments, we tested several values of *κ*, and obtained the best results with *κ *= 8. Small oligomers of length one and two could be omitted. This resulted in an equal classification rate while the computational time was significantly reduced. Therefore, we adapted *σ*_3_,...,*σ*_8_. The regularization parameter *C *of the SVM was adapted using one-dimensional grid-search. We considered grid points {0.1·*i *| 1 ≤ *i *≤ 50}. For the locality improved kernel, a three-dimensional grid-search and 5-fold cross-validation was used for the three parameters *C *(regularization parameter of the SVM), *l *and *d*. We considered *C *∈ {0.002·*i *| 1 ≤ *i *≤ 10} and *l*, *d *∈ {*i *| 1 ≤ *i *≤ 6}.

For the Markov chain model, the order *n *and the value of the pseudocount *c*_*pseudo *_had to be adapted. We used grid-search over the values *c*_*pseudo *_∈ {0.2·*i *| 1 ≤ *i *≤ 10} and *n *∈ {*i *| 0 ≤ *i *≤ 5} in a five-fold cross-validation scenario.

The final values for the smoothing parameters *σ*_3_,...,*σ*_8 _of the combined oligo kernel and the regularization parameter C of the SVM are given in Table 2. The smoothing parameters show that the positional uncertainty increases with oligomer length. An exception is the parameter *σ*_4 _which is very small and thus, on the level of tetramers, the optimized kernels used Gaussians that were narrow peaks and virtually just counted exact matches. However, there was a considerable increase in *σ*_*i *_for *i *≥ 5. On the level of pentamers and longer fragments, matching subsequences could shift by several nucleotides and still contribute to the similarity of two sequences. Note that a *σ*_*i*_-value of 2.5 implies that a subsequence shifted by three nucleotides still has 70 percent of the contribution of an exact match in the kernel function (2). Table 3 shows the statistics of the final hyperparameters for the locality improved kernel and the Markov chain model. The order of the Markov chain was about two. One reason for the low order is the limited amount of training data which does not allow for estimation of too many model parameters.

**Table 2:** The adapted parameters for the combined oligo kernel

	*σ*_3_	*σ*_4_	*σ*_5_	*σ*_6_	*σ*_7_	*σ*_8_	*C*
mean	6.61	0.39	15.15	547.93	914.27	841.61	2.8
25% quantile	5.91	0.32	5.19	119.11	914.27	914.27	2.3
median	6.39	0.43	18.27	808.02	914.27	914.27	2.5
75% quantile	7.18	0.43	20.31	914.27	914.27	914.27	3.0

This table shows the adapted *σ*_*i *_for the combined oligo kernel.

**Table 3:** Final hyperparameter configurations for locality improved kernel and Markov chain model

	locality improved			Markov chain model	
	*C*	*l*	*d*	*n*	*c*_pseudo_
mean	0.01	1.54	4.54	2.3	0.33
25% quantile	0.002	1	3	2.0	0.2
median	0.002	2	4	2.0	0.2
75% quantile	0.02	2	6	2.75	0.4

The Results for the Final Hyperparameter Configurations over the 50 Partitions for the Locality Improved Kernel and the Markov Chain Model.

#### Identification of relevant features for classification

To shed light on relevant features which were used by the SVM classifier, visualization techniques as described in [22] were employed [see Methods]. For this purpose, SVMs with oligo kernels using oligomers of length three, four, five and six were employed with the SVMs. These kernels resulted in an inferior classification rate while providing well-interpretable parameters.

**Figure 2:**
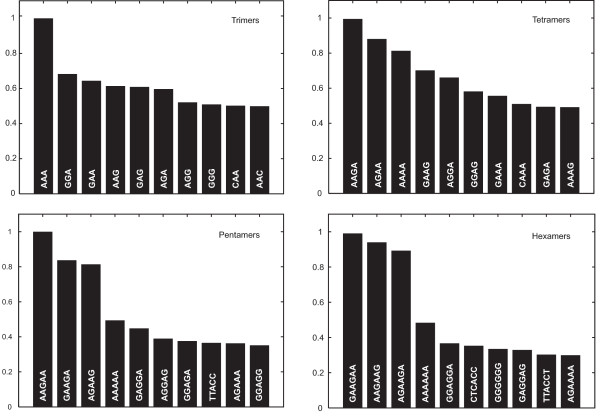
**Oligomer ranking**. The ten most important oligomers for discrimination based on trimers, tetramers, pentamers and hexamers are shown. The heights of the bars correlate to the average norm of the corresponding *K*-mer weight function and was scaled to yield an unit maximum.

In order to extract the most important oligomers for the kernel-based ESE prediction, the oligomer-specific weight functions of the discriminant were calculated. The ten most important *K*-mers, *K *= {3,4,5,6}, were identified and displayed in a bar graph in Figure 2. The height of each bar correlates to the average norm of the corresponding *K*-mer weight function and was scaled to yield an unit maximum. For the oligomers shown in Figure 2, one can identify a group of motifs which is most prominent. These are the motifs which occur in the purine-rich enhancers, as for example GAGGAG or GAAGAA. These motifs are represented by several of the important oligomers shown in Figure 2.

**Figure 3:**
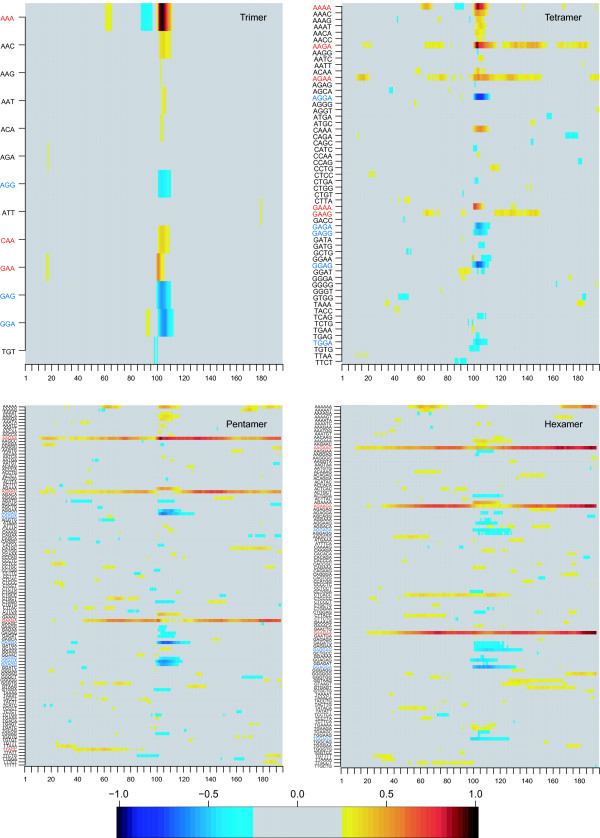
**Image matrix of discriminative weight functions**. The image was derived from the trained classifiers based on the trimer, tetramer, pentamer or hexamer kernel. Each of the lines shows the values of one specific weight function obtained from an average over 50 runs. Each of the 200 columns corresponds to a certain sequence position. By construction, the exonic splicing enhancer motif starts at position 100. For noise reduction all matrix elements below 0.25 have been zeroed.

Figure 3 exhibits the positions in the sequences at which relevant features are located. High positive (negative) values correspond to relevant features for discriminating positive (negative) examples. One can see that oligomers that were important for the classification were often located in the middle of the sequences. This seems to be consistent as the exonic splicing enhancers are, by construction, always located in the middle of the training data [see Methods] and these oligomers are contained in a large group of ESEs known as purine-rich enhancers containing repeated GAR (GAA or GAG) trinucleotides. Additionally, it can be inferred that oligomers which were important for the classification of positive examples (red in Figure 3) are mostly composed of purines but with a higher amount of adenine. In the negative examples (blue in Figure 3) this is inverted and guanine was more frequently present. This correlates to the fact that the most frequent middle-motifs in negative examples were GGAGGA or GAGGAG. In positive examples GAAGAA or AAGAAG were most frequent. To check whether the classifier simply did not recognize these differences, we examined the classification performance using only the frequencies of the hexamers in the middle of the sequence [see Methods]. Using only this information we obtained a classification rate of 66.8% showing that other features must play an important role for classification as well.

#### Classification performance

The classification performances of the different methods are shown in Table 4. The table gives the mean values as well as 25, 50, and 75 percent quantiles over the 50 partitions of the classification rate on the test set (accuracy), specificity, sensitivity, and Matthews correlation coefficient [25]. Specificity is defined by *TN*/(*TN *+ *FP*), sensitivity by *TP*/(*TP *+ *FN*), and Matthews correlation coefficient by

$$\frac{TP\times TN-FP\times FN}{\sqrt{\left(TP+FP)(TP+FN)(TN+FP)(TN+FN\right)}}$$

where TP, TN, FP, and FN denote the true positive, true negative, false positive and false negative rates, respectively. For clarification, the notation "true negative" denotes the fraction of negative examples that are classified as negatives, whereas "false negative" indicates the fraction of positive examples that are incorrectly classified as negatives. Using SVMs with the combined oligo kernel, the best classification rates could be achieved. The accuracy of the SVM with optimized combined oligo kernel was significantly better than the accuracy of the SVM with locality improved kernel (paired Wilcoxon rank sum test, *p *< 0.001) as well as the accuracy of the Markov chain model (paired Wilcoxon rank sum test, *p *< 0.001). The SVM with combined oligo kernel achieved a classification rate of 90.74%, the SVM with locality improved kernel achieved a classification rate of 70% and the Markov model achieved a classification rate of 68.42%. We did not test the SVM classifiers using external test data, because they were only trained on exonic data.

**Table 4:** Classification results for motif-oriented data using different kernels

	accuracy	specificity	sensitivity	correlation
SVM, combined oligo kernel	90.74%	96.04%	82.09%	78.93%
25% quantile	90.45%	95.4%	81.16%	78.42%
median	90.82%	96.04%	82.09%	79.23%
75% quantile	91.22%	96.62%	83.25%	79.93%

SVM, locality improved kernel	70.00%	92.45%	33.36%	32.43%
25% quantile	69.16%	89.06%	24.45%	30.73%
median	69.88%	91.43%	38.56%	32.33%
75% quantile	70.93%	96.49%	41.37%	34.49%

Markov chain model	68.42%	79.26%	50.71%	31.44%
25% quantile	67.98%	76.17%	50.95%	30.66%
median	68.29%	77.61%	53.7%	31.67%
75% quantile	68.89%	80.57%	55.02%	32.64%

The mean values, 25 percent quantile, median and 75 percent quantile of the accuracy, specificity, sensitivity and Matthews correlation over 50 trials are given.

Due to this it would not have made sense to test with intronic or intergenic data sets. In contrast, for the neutralized data the testing with external data sets was necessary, because the idea behind the data is that a classifier can extract exon-specific features. This needs to be tested using external data such as, for example, introns or intergenic regions.

**Figure 4:**
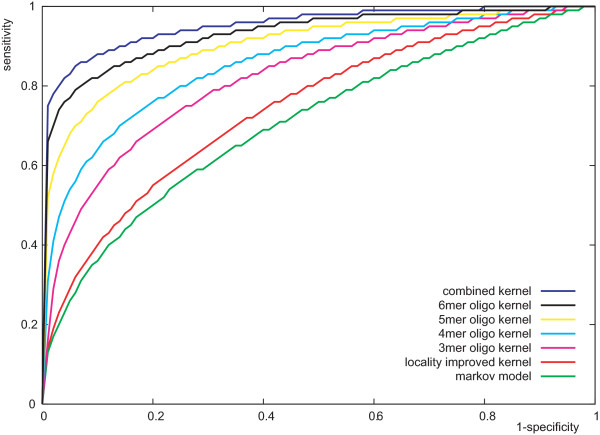
**Receiver operating characteristics (ROC) for the classifiers**. Median ROC curves of the classifiers based on 50 trails are shown.

In Figure 4, the receiver operating characteristics (ROCs) of the classifiers are shown. For the SVMs, the curves were obtained by simply varying the threshold parameter *b *[26]. For the Markov chain model, a threshold parameter *b *was introduced and adjusted, that is, a sequence was classified based on the sign of ln $P^{M^{+}}$ (**s**) - ln $P^{M^{-}}$ (**s**) + *b*. Each curve in Figure 4 corresponds to the median of the 50 trials (similar to the attainment surfaces described in [27]). The superior performance of the SVM with combined oligo kernel was also supported by the receiver operating characteristics in Figure 4, while the Markov chain model showed the worst performance. The SVM with 6-mer oligo kernel performed only slightly worse than the SVM with combined oligo kernel indicating that the hexamers are important for this classification problem.

#### Consistency of results

As the ESE pattern in the middle of the motif-oriented data is in the vicinity of the splice site, the intronic part can be suspected to be the main contributor to the classification rate. However, on average, the middle of the motif-oriented data, i.e., the ESE patterns in these positive examples, has a distance of 40 bp to the splice site. Thus, the larger part of the examples is exonic. Additionally, the fraction of examples that is intronic differs between the various training examples and is thus no fact the classifier can rely on. Furthermore, the analysis of the image matrices (Figure 3) confirms the importance of the middle motifs as they are indicated there as important for classification. In order to support this interpretation, an experiment was conducted where the position of negative examples to the vicinity of splice sites (as it was the case for positive examples). Training an SVM with these data resulted in classification rates of about 80% which demonstrates that the classifier uses more than exon/intron distinctions for its decision.

In order to investigate the influence of the central motif relative to its surround on classification performance, another set of training examples was extracted in order to train an SVM. In these sets of positive and negative examples, any of the 4096 possible hexamers was accepted as a middle motif for a positive or negative example. Predictably, the classification rate dropped by 6% although this was not as strong a drop as might be expected. One reason is conceivable: only a subset of all 4096 possible hexamers actually occurs in the training data since the number of training examples had to restricted due to the unfavorable scaling behavior of SVMs w.r.t. the number of training examples. If the classifier is to learn a reliable decision function, each middle motif should occur not only once but several times both in the positive and negative training examples. It is therefore easy to see that the amount of needed training data grows strongly with the number of used middle motifs. The number of training examples that could be used (due to the restrictions of the SVM) must be considered to be far too small in the case of 4096 middle motifs. If all possible training examples obtained using the 4096 possible hexamers as middle motifs can be used, we expect a much stronger drop in performance.

### Comparison with ESEfinder

In order to compare our approach to a current state of the art in ESE detection, we choose ESEfinder [12] as a reference. We used ESEfinder on all positive and negative examples that were taken to train and test our SVM classifier. We counted only recognized motifs in the middle of sequences in this procedure, as we placed the true motif in the middle of the test sequences. Any motif in the vicinity of the potential ESE in the middle of the sequence can be the middle of another sequence due to our selection method. Of course, because of the fact that ESEfinder is based on position weight matrices (PWMs) it is hard for the classifier to distinguish between positive and negative examples. As expected, ESEfinder misclassified many of the sequence motifs and found a considerable number of "ESEs" in the negative examples. We only obtained an overall classification performance of 44% using ESEfinder. As a consequence, we were interested in observing how well ESEfinder performs when classifying only the positive examples. We obtained a true positive rate of 39%. This might be due to the fact that our middle-motifs were hexamers determined by RESCUE-ESE [10] from which not all were represented by the PWMs used in ESEfinder. The low true positive rate might have two explanations, either ESEfinder might need an update of the matrices or not all hexamers identified by RESCUE-ESE might be ESEs.

### General discussion

The best-case scenario for the proposed SVM approach would be the exclusive usage of biologically verified training examples. For the required number of training examples, this is impractical and is likely to remain impractical in the near future. We have shown that, using unverified training data, a meaningful decision function can be learned. Furthermore, arguments for the correlation of this decision function to ESE activity were presented. Based on the experiments described in this paper, we see the value of our method in its fundamental suitability for this classification problem and its ability to incorporate expert knowledge into the training data generation process. This can be done by choosing appropriate biologically verified heuristics for selecting training data. As a consequence, not every positive example will correspond to a "true" ESE (the inverse of course holds for the negative examples). However, by virtue of the used heuristics a significant over-representation of ESEs in the positive training examples as well as a corresponding under-representation in the negative training examples is reasonable to assume. Therefore, we do not expect the SVM to perform perfectly but to have a classification rate significantly above chance. Just as other approaches [12], the SVM will produce incorrect predictions, although we are confident that new insights into the splicing process can be used in a straightforward way to improve the already favorable results still further.

## Conclusion

We successfully trained and used SVMs with special sequence based kernels for the detection of exonic splicing enhancers. The main problem was the choice of training examples due to the small amount of annotated exonic splicing enhancers in the literature. As we did not obtain good results using our first approach, the neutralized data, we developed a new method for choosing training and test examples. This includes extracting motifs from the exons as well as filter them out according to heuristics based on known properties of ESEs. Negative examples were extracted from the middle of longer exons, where presumably no ESEs are located in order to have a set of reliable negative examples. Initial tests showed that these sequences were useful for training an SVM classifier, leading to good results. From the different tested kernels, the best results were obtained using the combined oligo kernel with 90.74% accuracy, a specificity of 96.04% and a sensitivity of 82.09%. From a machine-learning point of view, an SVM is a linear classifier in a feature space and the quality of the SVM is to nearly 100 percent based on the used kernel function realizing a scalar product or similarity measure in that feature space [28]. Thus, for obtaining such favorable results, an appropriate kernel in the form of the combined oligo kernel was a necessary prerequisite for successful classification. As can be seen from the results with locality-improved kernel, using another kernel leads to inferior results. To check the benefit from using SVMs we applied a Markov model to the data which resulted in a significantly lower classification rate (68.42% accuracy). The parameters of the oligo kernel were well interpretable and gave information that longer oligomers can shift in the sequence by several bases. Additionally, the oligo kernels can be visualized, presenting important oligomers for ESE classification. We showed that our SVM approach compares favorably to a well-known state of the art method (ESEfinder).

In the future, we would like to create a web-based version of the program in order to make it usable for the research community. Additionally, it may be useful to integrate the enhancer prediction into a splice site prediction program, as it was already done for Arabidopsis thaliana in [29].

## Methods

In this section, oligo kernels for the analysis of biological sequence data are described. Furthermore, the locality improved kernel which we considered for comparison is described and Markov chain models are introduced as an alternative classification method. Additionally, the methods for choosing the training and test data are presented.

### Classification with SVMs

We consider *L*_1_-norm *soft margin support vector machines *(SVMs) for binary classification [14-16]. Let (*x*_*i*_, *y*_*i*_), 1 ≤ *i *≤ *l*, be *consistent *training examples, where *y*_*i *_∈ {-1, 1} is the label associated with input pattern *x*_*i *_∈ *X*. The main idea of SVMs is to map the input patterns to a feature space *F *and to separate the transformed data linearly in *F*. The transformation *Φ *: *X *→ *F *is implicitly done by a kernel *k *: *X *× *X *→ ℝ, which computes a scalar (inner) product in the feature space efficiently, that is, *k*(*x*_*i*_, *x*_*j*_) = ⟨*Φ*(*x*_*i*_), *Φ*(*x*_*j*_)⟩.

### Oligo Kernels

For oligo kernels [22,24,30], the feature space can be described using *oligo functions*. These code for occurrences of oligomers in sequences with an adjustable degree of positional uncertainty. In existing methods, they provide either position-dependent [31] or completely position-independent representations [32]. For an alphabet A and a sequence s, which contains *K*-mer *ω *∈ $A^{K}$ at positions $S_{\omega }^{s}=\{p_{1},p_{2},...\}$, the oligo function is given by

(1) $$\mu_{\omega }^{s}(t)=\sum_{p\in S_{\omega }^{s}}\exp\left(-\frac{1}{2\sigma^{2}}{\left(t-p\right)}^{2}\right)$$

for *t *∈ ℝ. The smoothing parameter *σ *adjusts the width of the Gaussians centered on the observed oligomer positions and defines the degree of position-dependency of the function-based feature space representation. While small values for *σ *imply peaky functions, large values imply flatter functions. For a sequence s the occurrences of all *K*-mers contained in $A^{K}$ = {*ω*_1_, *ω*_2_,...,*ω*_*m*_} can be represented by a vector of *m *oligo functions. This yields the final feature space representation $\Phi (s)={\left[\mu_{\omega_{1}}^{s},\mu_{\omega_{2}}^{s},...,\mu_{\omega_{m}}^{s}\right]}^{T}$ of that sequence. A kernel function is build to compute the dot product in the feature space efficiently, in order to make it suitable for learning. The inner product of two sequence representations *Φ*_*i *_and *Φ*_*j*_, corresponding to the oligo kernel *k*(**s**_*i*_, **s**_*j*_), can be defined as

(2) $$\begin{array}{lll} \langle \phi_{i},\phi_{j}\rangle & = & \int \phi_{i}(t)\cdot \phi_{j}(t)\text{d}t\\ & = & \sum_{\omega \in A^{K}}\sum_{p\in S_{\omega }^{i}}\sum_{q\in S_{\omega }^{j}}\exp\left(-\frac{1}{4\sigma^{2}}{\left(p-q\right)}^{2}\right)\\ & = & k(s_{i},s_{j}) \end{array}$$

writing *Φ*_*i *_for $\Phi_{s_{i}}$. In order to improve comparability between sequences of different lengths, we compute the normalized oligo kernel

(3) $$\tilde{k}(s_{i},s_{j})=\frac{k(s_{i},s_{j})}{\sqrt{k(s_{i},s_{i})k(s_{j},s_{j})}}.$$

**Figure 5:**
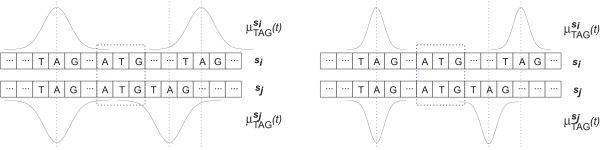
**Effect of the smoothing parameter**. Example of two sequences *s*_*i *_and *s*_*j *_and the corresponding oligo functions for *ω *= TAG for small (left) and large (right) smoothing parameter *σ*_3_. On the left-hand side, it can be seen that the larger *σ*_3 _results in Gaussians that are still overlapping although the motif TAG is shifted in the two sequences. On the right-hand side, the shifted TAG motifs do not increase the kernel function due to the strongly peaked Gaussian.

From the formula for the oligo kernel, the function of the parameter *σ *becomes clear, see also Figure 5. For *σ *→ 0 only oligomers which occur at the same positions in both sequences contribute to the sum. In general, it is not appropriate to represent oligomer occurrences without positional uncertainty. This would mean zero similarity between two sequences if no *K*-mer appears at *exactly *the same position in both sequences. For *σ *→ ∞ position-dependency completely disappears. In this case all oligomers which occur in both sequences contribute equally to the sum, regardless of their distance and the oligo kernel becomes identical to the spectrum kernel [32].

### Combined Oligo Kernel

Meinicke et al. already showed that it is beneficial to employ combinations of oligo kernels that consider oligomers of different lengths [22]. The *κ*-*combined oligo kernel*

(4) $${\tilde{k}}_{\kappa \text{-combined}}(s_{1},s_{2})=\frac{1}{\kappa }\sum_{i=1}^{\kappa }{\tilde{k}}_{i}(s_{1},s_{2})$$

was introduced, where the subscript *i *indicates that the normalized oligo kernel ${\tilde{k}}_{i}$ is defined on the oligomers of length *i*. The level of position-dependency can be controlled for each oligomer length individually using *κ *parameters *σ*_1_,..., *σ*_*κ*_.

### Visualization of Oligo Kernels

The oligo kernel can easily be visualized using the weight vector as a vector-values function arising from a linear combination of the feature space representation. With the learned parameters *α*_*i *_we can construct the vector-valued weight function of the discriminant as

(5) $$w(t)=\sum_{i=1}^{n}\alpha_{i}{\left[\mu_{1}^{i}(t),\mu_{2}^{i}(t),...,\mu_{m}^{i}(t)\right]}^{T},$$

with $\mu_{\omega }^{s}(t)$ as in equation (1). This is a curve in the *m*-dimensional space of oligomers. For each of the *m *components we have a linear combination of the oligo functions where the weights *α*_*i *_determine the contribution from each of the *n *training sequences. Due to the fact that the feature space vector can be represented as a vector of functions, all discriminative weight functions *w*_*i *_may be discretized and stored in a matrix which may be visualized as a bitmap image using color. Here, we used discrete sequence positions *t *∈ {0,...,ℓ}, with ℓ = sequence-length -*K*, resulting in an *m *× ℓ matrix

(6) *W *= [*w*(*t*_1_), *w*(*t*_2_),...,*w*(*t*_*l*_)].

when *m *is the number of oligomers. For noise reduction all values between 0.25 and -0.25 were set to zero and rows which were totally zero were excluded.

In order to reduce the complexity of the interpretation, the analysis can be restricted to the most important oligomers. Therefore, the component weight functions of *w*(*t*) = [*w*_1_(*t*), *w*_2_(*t*),...,*w*_*m*_(*t*)]^*T *^can be ranked according to their *L*_2_-norm

(7) $$\begin{array}{cc} N_{i}=\sqrt{\int w_{i}{\left(t\right)}^{2}dt}, & i=1,...,m \end{array}$$

The norm was approximated using the Euclidean norm of discretized oligo functions. Higher norms indicate a more important role in discrimination and the selection of corresponding weight functions helps to focus on important oligomers.

### Locality improved kernel

For comparison, we consider the locality improved kernel [15,23] which counts matching nucleotides and considers local correlations within windows of length 2*l *+ 1. For two sequences **s**_*i*_, **s**_*j *_of length *L *the locality improved kernel is given by

(8) $$k_{\text{locality}}(s_{i},s_{j})=\sum_{p=1}^{L}{\left(\sum_{t=\max(1,p-l)}^{\min(L,p+l)}v_{t+l-p}\cdot {\text{match}}_{t}(s_{i},s_{j})\right)}^{d}$$

Here, match_*t *_(**s**_*i*_, **s**_*j*_) = 1, if **s**_*i *_and **s**_*j *_have the same nucleotide at position *t *and zero otherwise. The weights *v*_*t *_give us the possibility to emphasize regions of the window which are of special importance. In our experiments they are fixed to *v*_*t *_= 0.5 - 0.4|*l *- *t*|/*l*. The hyperparameter *d *determines the order to which local correlations are considered. The locality improved kernel can be considered as a special form of a *polynomial kernel*, where only a weighted subset of *monomers *is considered [15].

### Markov chain model

As a baseline classifier, we look at simple Markov models of the positive and negative sequences, see [33] for an introduction. We apply *inhomogeneous Markov chains*, also referred to as *weight array matrix models*. Given a Markov chain *M *of order *n *over an alphabet A for strings of a fixed length *l *(cf. [[33], Section 4.4.2] and [34]), the likelihood of a sequence is given by

(9) $$\begin{array}{r} P^{M}(s)=p_{1}^{M}(s_{1})\cdot P_{2}^{M}(s_{2}|s_{1})\cdot ...\cdot P_{n}^{M}(s_{n}|s_{1},...,s_{n-1})\\ \cdot \prod_{i=n+1}^{l}P_{i}^{M}(s_{i}|s_{i-n},...,s_{i-1}). \end{array}$$

The conditional probabilities $P_{i}^{M}$ are the $\frac{\left|A{|}^{n+1}-|A\right|}{|A|-1}+(l-n)|A{|}^{n+1}$ parameters of the model and are estimated from the frequencies in the training data plus a *pseudocount c*_pseudo _(cf. [[33], Section 4.3.1]). Let *M*^+ ^and *M*^- ^be the Markov chain models built from the positive and negative examples in the training data, respectively. A sequence s is classified based on the sign of *ln*$P^{M^{+}}$ (**s**) - *ln*$P^{M^{-}}$ (**s**). Our simple Markov chain model has only two hyperparameters, its order *n *and the value of the pseudocount *c*_*pseudo*_. The latter serves as a regularization parameter.

### Motif-oriented classification

The classification performance considering only the frequencies of the motifs in the middle of the sequences was calculated using the same data partitionings in training and test data as in the classification using SVM or Markov model. For each partition of the data the frequencies of the different motifs in the middle of the training sequences were counted. Now, for each test sequence, we extracted the middle-motif and decided whether the test sequence was positive or negative with the previously determined motif numbers in the training set. Therefore, if a certain motif is overrepresented in the positive training examples the test sequence is classified as being positive, otherwise it is classified negative.

### Data sets

#### Neutralization

Neutralization is a strategy to generate transformed sequences from exons which still code for the same amino acid sequence and maintain the overall composition of the original exons. Three criteria have to be met while the exons are transformed. Firstly, the neutralized sequence codes for the same protein. Secondly, a codon should not be used more frequently to represent a particular amino acid than in the original set. Thirdly, the frequencies of the dinucleotide occurrence should be retained. For the detailed algorithm of the neutralization method, we refer to the original literature [17].

#### Motif-oriented data-extraction method

**Figure 6:**
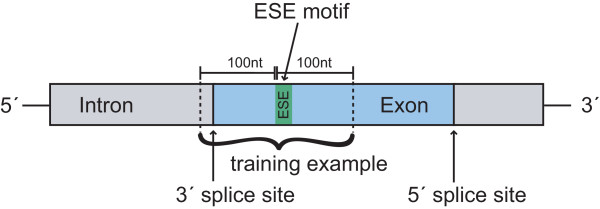
**Schematic presentation of the data-extraction method**. Sequences are extracted locally around potential ESE motifs and are then declined or accepted as positive examples depending on whether they fulfill certain criteria. First of all, a potential positive example has to be close to a splice site. Secondly, the exon from which the positive example is extracted has to be a weak exon and thirdly, the region in which the positive example is located has to be single-stranded. Each training sequence has a length of 200 bases.

The basic problem with ESE classification is the small amount of verified data from the literature or databases which can be used for training and testing machine learning approaches. Because the motifs of the ESEs are known, the positive examples can be extracted from the exons but not every motif found in this way is a real ESE. This leads to unreliable positive examples. We developed a new data-extraction scheme (see Figure 6) where the sequence located around a potential ESE is extracted. A surrounding of 200 bases was considered as sufficient.

**Figure 7:**
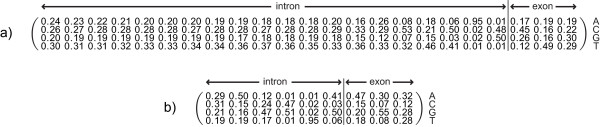
**Position weight matrix for the 3' and the 5' splice site**. The rows represent the bases A, C, G and T. Each column stands for one sequence position in the consensus sequence. Each entry represents the normalized number of occurrences of the base at that position. Each row is added to 1. For the 3' splice site a surrounding of 23 bases and for the 5' splice site a surrounding of 9 bases is considered important.

To deal with the drawback of unreliable positive examples, each extracted sequence has to meet several criteria which increase the possibility of a motif being an ESE. First of all, only potential ESE motifs in the vicinity of the splice sites are used because it is stated in the literature that ESE sequences are not active far away from the splice sites [19,20]. Therefore, only motifs with distances of less than 100 nucleotides from the splice sites are considered as potential ESE sequences. Furthermore, as claimed in [3,10], ESE motifs can compensate for the presence of "weak" (non-consensus) 3' or 5' splice sites of exons. These exons are under a much higher selective pressure to retain ESE motifs and therefore they often contain a higher amount of exonic splicing enhancers. To include this fact into our training data, we generated position-specific weight matrices (PWMs) for both the 3' splice site and the 5' splice site. We extracted all annotated splice sites from the Vega database [18]. For the 3' splice site, we took 20 bases of the intron and 3 bases of the exon to take the pyrimidine-rich sequence into account. For the 5' splice site, 3 bases of the exon and 6 bases of the intron were considered as splice site. These differences result from the known design of the splice sites, including the pyrimidine-rich sequence into the 3' splice site (see Figure 1). Creating the PWMs, we obtained a 4 × 23-matrix for the 3'splice site and a 4 × 9-matrix for the 5' splice site, which are shown in Figure 7.

Using these PWMs, a score was assigned to every splice site. The score assigned by a PWM to a substring is defined as $\sum_{j=1}^{N}log\left(\frac{p_{ij}}{b_{i}}\right)$, where *p*_*ij *_is the probability of observing symbol *i *at position *j *of the motif and bi is the probability *b*_*i *_of observing that symbol in the background model. For the background model, we considered all bases as equally represented. Those splice sites with a score among the lower 25% of scores were classified as a weak splice site and those among the upper 25% were classified as strong. Then, only motifs in the vicinity of "weak" exons were considered as being reliable training examples.

Third, RNA binding proteins recognize RNA in a sequence-specific manner where the secondary structure of the RNA plays a role [21]. Binding sites as ESE sequences are often located in single stranded regions. A motif in a double-stranded region has been shown experimentally to have a strong negative correlation with the binding affinity [35] or even abolishes protein-binding [36,37]. Therefore, we calculated energy parameters to characterize the single-strandedness of a substring in an RNA sequence. For characterization of single-stranded regions, we used a parameter *EF*_*a,b *_described in [21] giving the expected fraction of bases in the substring from position *a *to position *b *that do not form base pairs. *EF*_*a,b *_is calculated as

(10) $$EF_{a,b}=1-\frac{\sum_{i=a}^{b}\sum_{j=1}^{L}p_{i,j}}{b-a+1}$$

with *L *being the length of the RNA sequence and *p*_*ij *_giving the possibility that base *i *and *j *are paired. This parameter can be calculated with the help of RNAfold [38]. Using *EF*_*a,b *_> 0.6, only potential ESEs located in single stranded regions were considered as positive examples.

## Abbreviations

ESE: exonic splicing enhancer; SVM: support vector machine; PWM: position-weight matrix; ROC: receiver operating characteristics.

## Authors' contributions

BM conceived the study, created the data sets, implemented the tests and wrote the manuscript. AG gave helpful suggestions for the machine learning part. SS provided guidance and helped to finish the manuscript. AH supervised the whole project. All authors read and improved the manuscript.
